# Carvacrol Modulates the Hippocampal Prostaglandin–Cytokine Axis in LPS-Induced Neuroinflammation

**DOI:** 10.3390/biomedicines14071428

**Published:** 2026-06-24

**Authors:** Ayse Ozkan, Seda Demir

**Affiliations:** Department of Physiology, Faculty of Medicine, Izmir Bakırçay University, Izmir 35665, Türkiye; sedademir95@hotmail.com

**Keywords:** carvacrol, lipopolysaccharide, neuroinflammation, prostaglandin E_2_, Y-maze, open-field test

## Abstract

**Objective:** Neuroinflammation contributes to cognitive impairment across neurodegenerative disorders. Prostaglandins (e.g., PGE_2_, PGD_2_, PGF_2_α) and pro-inflammatory cytokines (TNF-α, IL-1β) are key mediators of lipopolysaccharide (LPS)-induced hippocampal dysfunction. Carvacrol (CRV), a monoterpenic phenol with anti-inflammatory and antioxidant properties, may mitigate these effects, but its impact on hippocampal prostaglandin profiles is not well-defined. **Methods:** BALB/c mice were randomly assigned to Control (PBS; *n* = 7), LPS (1 mg/kg, i.p.; *n* = 10), or LPS + CRV (50 mg/kg, p.o.; *n* = 7). Body weight was tracked daily for 7 days; rectal temperature was measured once before behavioral testing and euthanasia. Locomotion/anxiety were assessed by the open-field test (OFT; average speed, total distance, freezing, mobility rate) using ToxTrac. Spatial recognition memory was evaluated in the Y-maze novel-arm task (novel-arm entries, duration, total entries, discrimination index [DI]). Hippocampal PGE_2_, PGD_2_, PGF_2_α, TNF-α, and IL-1β were quantified by ELISA. Data were analyzed by one-way ANOVA with Sidak’s post hoc test. **Results:** OFT measures did not differ among groups (*p* > 0.05), indicating no confounding locomotor deficits. In the Y-maze, LPS reduced novel-arm entries versus the Control (*p* = 0.0029), while LPS + CRV showed a nonsignificant increase versus LPS (*p* = 0.2406). Novel-arm duration differed among groups (*p* = 0.0033); LPS + CRV spent less time than LPS (*p* = 0.0128). Critically, DI showed a robust treatment effect (*p* < 0.0001): LPS markedly impaired DI versus the Control (*p* < 0.0001), and CRV significantly improved DI versus LPS (*p* < 0.0001). Biochemically, LPS elevated hippocampal PGE_2_ (*p* < 0.0001) and PGF_2_α (*p* = 0.0040); CRV normalized PGE_2_ (*p* < 0.0001) but not PGF_2_α (*p* = 0.2656). PGD_2_ was unchanged. LPS increased TNF-α and IL-1β (both *p* < 0.0001), and CRV significantly reduced both versus LPS (both *p* < 0.0001). **Conclusions:** Acute LPS provokes prostaglandin- and cytokine-driven hippocampal inflammation with associated recognition memory deficits. Carvacrol attenuates cognitive impairment and suppresses hippocampal PGE_2_, TNF-α, and IL-1β, supporting a mechanism involving modulation of the prostaglandin–cytokine axis. These findings highlight CRV as a candidate adjunct for inflammation-associated cognitive dysfunction.

## 1. Introduction

Neuroinflammation is increasingly recognized as a major contributor to the pathogenesis of neurodegenerative and neuropsychiatric disorders, including Alzheimer’s disease, Parkinson’s disease, multiple sclerosis, and depression [1]. Activated microglia and astrocytes release pro-inflammatory cytokines and lipid mediators that impair neuronal function and synaptic plasticity, particularly within the hippocampus [2]. Among these mediators, tumor necrosis factor-alpha (TNF-α) and interleukin-1 beta (IL-1β) play central roles in amplifying inflammatory signaling and contributing to cognitive dysfunction [3,4]. Systemic administration of lipopolysaccharide (LPS), a potent toll-like receptor 4 (TLR4) agonist, is widely used to model neuroinflammation because it induces cytokine release, prostaglandin production, and hippocampal-dependent memory impairment [5]. Administering LPS leads to significant cytokine release, prostaglandin production, and behavioral disturbances that reflect inflammatory sickness behavior and cognitive decline in humans [6,7].

Prostaglandins are important lipid mediators involved in inflammatory signaling within the central nervous system [8]. Among them, prostaglandin E_2_ (PGE_2_) has been strongly associated with microglial activation, oxidative stress, and impaired synaptic plasticity in the hippocampus [9]. Increased prostaglandin signaling during inflammation contributes to neuronal dysfunction and cognitive deficits, partly through interactions with pro-inflammatory cytokines such as TNF-α and IL-1β [10]. Although prostaglandins are considered key components of neuroinflammatory cascades, their modulation in LPS-induced hippocampal dysfunction remains incompletely understood.

Carvacrol (5-isopropyl-2-methylphenol), a monoterpenic phenol present in oregano and thyme essential oils, has attracted attention because of its antioxidant, anti-inflammatory, and neuroprotective properties. Previous studies have shown that carvacrol suppresses NF-κB activation, inhibits COX-2 activity, and reduces pro-inflammatory cytokine production [11]. In addition, carvacrol has demonstrated beneficial effects in experimental models of oxidative stress, neurodegeneration, and cognitive impairment. However, limited information is available regarding its effects on prostaglandin-mediated neuroinflammatory pathways in the hippocampus.

Although the anti-inflammatory and neuroprotective effects of carvacrol have been investigated in previous experimental studies, its effects on hippocampal prostaglandin signaling during LPS-induced neuroinflammation remain insufficiently characterized. In particular, limited information is available regarding the simultaneous modulation of hippocampal prostaglandin mediators, including PGE_2_, PGD_2_, and PGF_2_α, together with pro-inflammatory cytokines in the context of cognitive dysfunction. Therefore, the present study aimed to investigate the effects of carvacrol on LPS-induced neuroinflammation and cognitive impairment in mice, with a particular focus on the prostaglandin–cytokine axis in the hippocampus. To this end, behavioral parameters related to locomotor activity and spatial memory were evaluated using the open-field and Y-maze tests, while biochemical analyses were conducted to quantify the hippocampal prostaglandin (PGE_2_, PGD_2_, and PGF_2_α) and pro-inflammatory cytokine (TNF-α and IL-1β) levels. We hypothesized that LPS administration would enhance hippocampal inflammatory signaling and cognitive dysfunction, whereas carvacrol treatment would attenuate these molecular and behavioral alterations. By integrating behavioral and biochemical findings, the present study provides a more comprehensive assessment of the potential modulatory effects of carvacrol on hippocampal neuroinflammation. In addition, elucidating the effects of carvacrol on the prostaglandin–cytokine axis may contribute to the development of novel adjunctive therapeutic approaches targeting inflammation-associated cognitive impairment in neurodegenerative and neuropsychiatric disorders.

## 2. Methods

### 2.1. Animals

A total of 24 BALB/c mice (male and female, 8 weeks old, 19–21 g) were obtained from the Laboratory Animal Research Center of Ege University (Izmir, Türkiye). Animals were housed under controlled environmental conditions (temperature: 21–24 °C; humidity: 55–65%; 12 h light/dark cycle) with ad libitum access to food and water. All experimental procedures were performed in accordance with the European Community Directive (86/609/EEC) and approved by the Institutional Animal Care and Use Committee of Ege University (Approval no: 2024-075, Date: 15 January 2025). All animal experiments and reporting procedures were conducted in accordance with the ARRIVE 2.0 (Animal Research: Reporting of In Vivo Experiments) guidelines.

Sample size was initially estimated by an a priori power analysis using G*Power 3.1 to achieve a statistical power of 0.90 at α = 0.05. Based on this calculation, a minimum of 10 animals per group was considered sufficient. However, due to unavoidable exclusions during the experimental process, the final group sizes were 7 for the Control group, 10 for the LPS group, and 7 for the LPS + CRV group.

### 2.2. Experimental Design

Mice were randomly divided into three experimental groups: Control (*n* = 7), LPS (*n* = 10), and LPS + Carvacrol (LPS + CRV, *n* = 7).

Control group: Mice received oral vehicle treatment for 7 consecutive days and a single intraperitoneal (i.p.) injection of phosphate-buffered saline (PBS; 10 mL/kg body weight) on day 6 (*n* = 7).

LPS group (LPS): Mice received oral vehicle treatment for 7 consecutive days and a single i.p. injection of lipopolysaccharide (LPS; 1 mg/kg; Escherichia coli serotype O111, Sigma-Aldrich, St. Louis, MO, USA) dissolved in PBS on day 6 (*n* = 10).

LPS + Carvacrol group (LPS + CRV): Mice received carvacrol (CRV; 50 mg/kg, p.o.) once daily for 7 consecutive days and a single i.p. injection of LPS (1 mg/kg) on day 6 (*n* = 7).

### 2.3. Drug Administration

Carvacrol (CRV; 50 mg/kg, p.o.; purity ≥ 98%, Sigma-Aldrich^®^, St. Louis, MO, USA) was dissolved in 2% Tween 80 and subsequently diluted with 0.9% saline. The solution was freshly prepared on each experimental day and administered once daily by oral gavage at a volume of 0.1 mL per 10 g body weight for 7 consecutive days. The selected CRV dose was based on previous studies demonstrating anti-inflammatory and neurobehavioral efficacy without significant impairment of locomotor activity or motor coordination [12].

Lipopolysaccharide (LPS; *Escherichia coli* strain O111, Sigma-Aldrich^®^, St. Louis, MO, USA) was administered as a single intraperitoneal injection at a dose of 1 mg/kg on experimental day 6. LPS was dissolved in phosphate-buffered saline (PBS) immediately before administration [13].

### 2.4. Physiological Measurements and Tissue Collection

Body weight was recorded daily throughout the 7-day experimental period to monitor general health and treatment effects. Rectal temperature was measured once, immediately before behavioral testing and prior to euthanasia, to assess systemic inflammatory responses. At the end of the experimental period, mice were deeply anesthetized and euthanized according to standard protocols [14]. Transcardial perfusion with saline was performed, and brains were rapidly removed. Hippocampal tissues were dissected and stored at −80 °C until biochemical analyses.

### 2.5. Biochemical Analyses

Hippocampal tissues were homogenized in cold buffer, and supernatants were collected by centrifugation. Levels of tumor necrosis factor-alpha (TNF-α) and interleukin-1 beta (IL-1β) were quantified using enzyme-linked immunosorbent assay (ELISA) kits (TNF-α: E-EL-M3063; IL-1β: E-EL-M0037, Elabscience, Wuhan, China) according to the manufacturer’s instructions. Prostaglandin concentrations (PGE_2_, PGD_2_ and PGF_2_α) were also determined using specific ELISA kits (PGE_2_: E-EL-0034; PGD_2_: E-EL-0066; PGF_2_α: E-EL-M1360, Elabscience, Wuhan, China). Absorbance was read spectrophotometrically, and concentrations were calculated from standard curves.

### 2.6. Behavioral Tests

#### 2.6.1. Open-Field Test

The open-field test (OFT) was used to evaluate locomotor activity and anxiety-like behavior [15]. Each mouse was placed in the center of an open-field arena (40 cm × 40 cm × 40 cm) and allowed to explore freely for 5 min. Behavioral parameters including average speed, total distance moved, total time frozen, and mobility rate were recorded and analyzed using the open-source video tracking software ToxTrac 2.70 [16].

#### 2.6.2. Y-Maze Test

Spatial working memory and exploratory preference were assessed with the Y-maze novel arm recognition task. The maze consisted of three identical opaque arms (5.5 cm wide, 35 cm long, 12.5 cm high) positioned at 120° from each other. The apparatus was placed in a quiet, evenly lit room (~25–30 lux at floor level). Mice were handled for 2–3 days before testing and acclimated to the testing room for ≥30 min prior to each session. The maze was cleaned with 70% ethanol and dried between trials to remove olfactory cues.

The task comprised a sample phase and a test phase. During the sample phase, one arm (designated the “novel” arm) was blocked, and mice were placed at the end of the start arm facing the center and allowed to explore the two open arms for 10 min. After an inter-trial interval of 60 min in the home cage, the test phase was conducted with all three arms open for 5 min. The blocked arm during the sample phase became the novel arm in the test phase; the other two were considered familiar arms. Arm designation (which arm was novel) was counterbalanced across animals and groups.

For the Y-maze test, behavioral recordings were independently evaluated by two blinded researchers to minimize observer bias and ensure scoring reliability. The following parameters were analyzed during the test phase: the number of novel arm entries, the duration spent in the novel arm (s), the total number of arm entries (as an indicator of locomotor activity), and the discrimination index (DI), which primarily reflects preference for the novel arm [17].

### 2.7. Statistical Analysis

Data were analyzed using GraphPad Prism 9 (GraphPad Software, San Diego, CA, USA). Normality was assessed using the Shapiro–Wilk test. For normally distributed data, one-way analysis of variance (ANOVA) followed by Sidak’s post hoc test was applied. Results were expressed as mean ± standard error of the mean (SEM), and *p* < 0.05 was considered statistically significant.

## 3. Results

### 3.1. Assessment of Locomotor and Exploratory Behavior in the Open Field Test

Supplementary Figure S1a illustrates the changes in body weight across all experimental groups throughout the study period. One-way ANOVA revealed no significant differences among groups (F(2,21) = 2.03, *p* = 0.1563, R^2^ = 0.1620). Sidak’s multiple comparisons test confirmed that neither the control and LPS groups (24.5 ± 0.6 g vs. 24.3 ± 0.6 g, *p* = 0.2372) nor the LPS and LPS + CRV groups (24.3 ± 0.6 g vs. 24.1 ± 0.6 g, *p* = 0.1704) differed significantly.

Similarly, no significant differences in body temperature were observed among the groups (Supplementary Figure S1b). One-way ANOVA showed no effect of treatment on body temperature (F(2,21) = 2.03, *p* = 0.1563, R^2^ = 0.1620). Post hoc comparisons revealed no differences between the control and LPS groups (36.03 ± 0.07 °C vs. 35.91 ± 0.07 °C, *p* = 0.2372) or between the LPS and LPS + CRV groups (35.91 ± 0.07 °C vs. 36.04 ± 0.07 °C, *p* = 0.1704).

**Figure 1:**
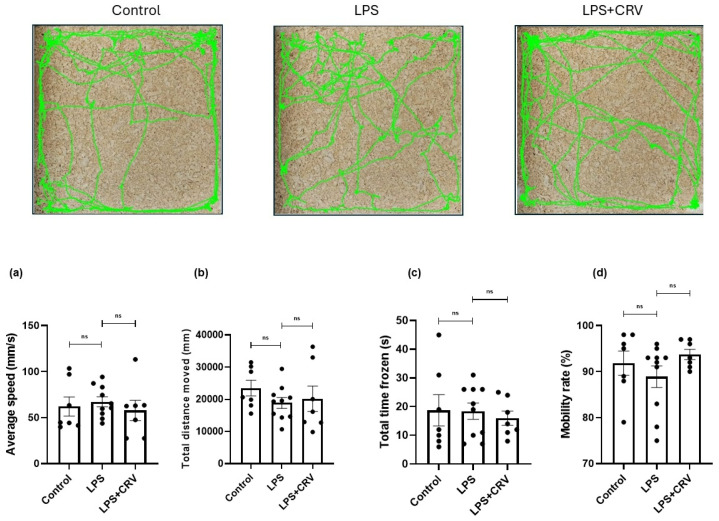
Effects of LPS and carvacrol on locomotor and exploratory behavior in the open-field test. (**a**) Average speed (mm/s), (**b**) total distance moved (mm), (**c**) total freezing time (s), and (**d**) mobility rate (%) measured during the 5-min open-field session. Data are presented as mean ± SEM. ns: non-significant.

In the open field test, treatment did not significantly affect locomotor performance (Figure 1). One-way ANOVA indicated no significant differences among groups for average speed (F(2,21), *p* > 0.05), and Sidak’s post hoc analysis confirmed the absence of group differences (Control vs. LPS: 62.01 ± 11.86 mm/s vs. 67.17 ± 11.86 mm/s, *p* = 0.8901; LPS vs. LPS + CRV: 67.17 ± 11.86 mm/s vs. 57.82 ± 11.86 mm/s, *p* = 0.6861) (Figure 1a).

Similarly, total distance moved did not differ significantly among groups (Control: 23,491 ± 3668 mm; LPS: 18,885 ± 3668 mm; LPS + CRV: 20,151 ± 3668 mm; *p* > 0.05) (Figure 1b).

There were also no significant differences in total freezing duration (Control: 18.71 ± 5.07 s; LPS: 18.40 ± 5.07 s; LPS + CRV: 16.00 ± 5.07 s; *p* > 0.05) (Figure 1c) or mobility rate (Control: 91.85 ± 3.12%; LPS: 88.90 ± 3.12%; LPS + CRV: 93.71 ± 3.12%; *p* > 0.05) (Figure 1d).

Collectively, these findings indicate that neither LPS nor carvacrol treatment significantly influenced locomotor or exploratory behavior, suggesting that subsequent cognitive assessments were not confounded by motor deficits.

### 3.2. Assessment of Spatial Recognition and Working Memory Performance in the Y-Maze Test

**Figure 2:**
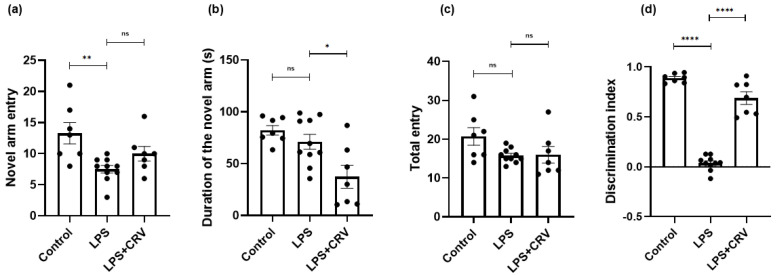
Y-maze novel arm recognition performance following LPS and carvacrol treatment. (**a**) Number of entries into the novel arm, (**b**) duration spent in the novel arm (s), (**c**) total arm entries, and (**d**) discrimination index (DI). Data are presented as mean ± SEM. * *p* < 0.05, ** *p* < 0.01, **** *p* < 0.0001, ns: non-significant.

In the Y-maze novel arm recognition task, significant group differences were observed in exploratory behavior and discrimination performance (Figure 2).

The number of entries into the novel arm differed significantly among the experimental groups (F(2, 21) = 6.706, *p* = 0.0056, R^2^ = 0.3897). Sidak’s multiple comparisons test revealed that LPS-treated mice made significantly fewer novel arm entries than the Control animals (7.50 ± 1.58 vs. 13.29 ± 1.58, *p* = 0.0029). Although the LPS + CRV group showed a moderate increase (10.00 ± 1.58), this difference was not statistically significant compared with the LPS group (*p* = 0.2406) (Figure 2a).

The time spent exploring the novel arm also varied significantly across groups (F(2, 21) = 7.611, *p* = 0.0033, R^2^ = 0.4203). No difference was detected between the Control and LPS groups (82.19 ± 11.12 s vs. 71.10 ± 11.12 s, *p* = 0.5510), whereas LPS + CRV mice spent significantly less time in the novel arm compared with the LPS group (37.45 ± 11.12 s vs. 71.10 ± 11.12 s, *p* = 0.0128) (Figure 2b).

General locomotor activity, assessed by total arm entries, did not significantly differ among the groups (F(2, 21) = 2.785, *p* = 0.0845, R^2^ = 0.2097). Sidak’s post hoc test indicated no significant differences between the Control and LPS (20.71 ± 2.25 vs. 15.80 ± 2.25, *p* = 0.0790) or between the LPS and LPS + CRV groups (15.80 ± 2.25 vs. 16.00 ± 2.25, *p* = 0.9951) (Figure 2c).

A highly significant treatment effect was detected for the discrimination index (F(2, 21) = 157.2, *p* < 0.0001, R^2^ = 0.9374). The DI was markedly reduced in the LPS group relative to the Controls (0.036 ± 0.051 vs. 0.888 ± 0.051, *p* < 0.0001), indicating profound impairment in spatial recognition memory. Carvacrol treatment significantly improved DI compared with the LPS group (0.688 ± 0.051 vs. 0.036 ± 0.051, *p* < 0.0001), partially restoring performance toward the control levels (Figure 2d).

Overall, these findings demonstrate that LPS administration impaired hippocampal-dependent spatial recognition memory, while carvacrol treatment attenuated this deficit, suggesting a potential neuroprotective effect against LPS-induced cognitive dysfunction.

### 3.3. Effects of LPS and Carvacrol on Hippocampal Prostaglandin Profiles

**Figure 3:**
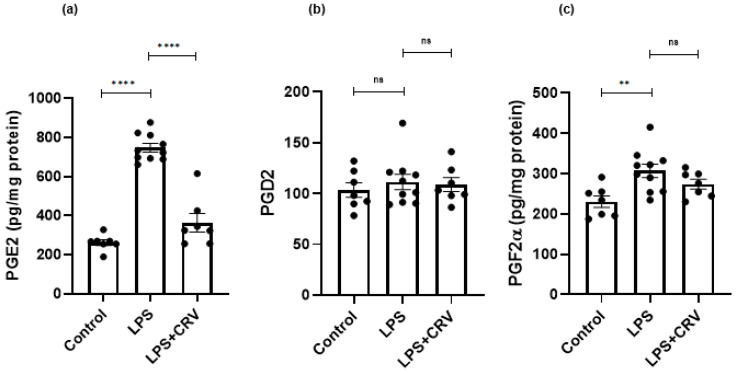
Hippocampal prostaglandin levels following LPS and carvacrol administration. (**a**) PGE_2_, (**b**) PGD_2_, and (**c**) PGF_2_α concentrations (pg/mg protein). Data are presented as mean ± SEM. ** *p* < 0.01, **** *p* < 0.0001, ns: non-significant.

Hippocampal PGE_2_ concentrations differed significantly among the experimental groups (F(2,21) = 80.87, *p* < 0.0001, R^2^ = 0.8851) (Figure 3a). Sidak’s post hoc analysis revealed that PGE_2_ levels were markedly elevated in the LPS group compared with the Control group (747.3 ± 41.3 pg/mg vs. 262.0 ± 41.3 pg/mg, *p* < 0.0001). Carvacrol treatment significantly attenuated this increase, reducing the PGE_2_ concentrations to 363.6 ± 41.3 pg/mg (*p* < 0.0001 vs. LPS).

In contrast, the hippocampal PGD_2_ concentrations did not significantly differ among groups (F(2,21) = 0.2950, *p* = 0.7476, R^2^ = 0.0273) (Figure 3b). Homogeneity of variances was confirmed by both Brown–Forsythe (*p* = 0.8405) and Bartlett’s (*p* = 0.7309) tests. Sidak’s post hoc analysis indicated no significant differences between the control and LPS groups (103.4 ± 10.4 pg/mg vs. 111.4 ± 10.4 pg/mg, *p* = 0.7004) or between the LPS and LPS + CRV groups (111.4 ± 10.4 pg/mg vs. 108.8 ± 10.4 pg/mg, *p* = 0.9608).

A significant group effect was observed for hippocampal PGF_2_α levels (F(2,21) = 6.230, *p* = 0.0075, R^2^ = 0.3724) (Figure 3c). Variance homogeneity was confirmed by Brown–Forsythe (*p* = 0.4843) and Bartlett’s (*p* = 0.3967) tests. Post hoc comparisons showed that the PGF_2_α concentrations were significantly higher in the LPS group than in the control group (306.7 ± 21.6 pg/mg vs. 230.5 ± 21.6 pg/mg, *p* = 0.0040). Carvacrol administration produced a moderate but non-significant reduction relative to LPS (273.8 ± 21.6 pg/mg vs. 306.7 ± 21.6 pg/mg, *p* = 0.2656).

Overall, these findings indicate that LPS administration induced a pronounced increase in hippocampal PGE_2_ and PGF_2_α levels, reflecting enhanced prostaglandin-mediated inflammatory activity, while carvacrol treatment effectively normalized the PGE_2_ but not PGF_2_α concentrations.

### 3.4. Hippocampal Pro-Inflammatory Cytokine Levels

**Figure 4:**
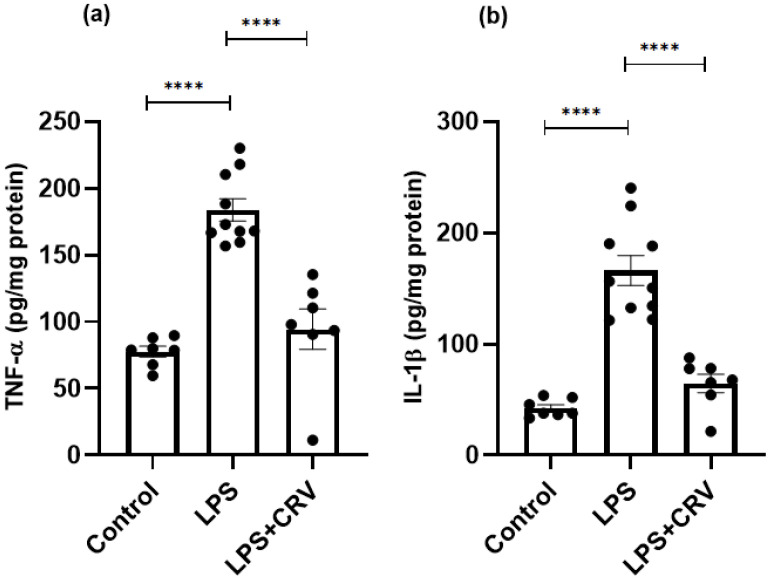
Hippocampal pro-inflammatory cytokine responses after LPS and carvacrol treatment. (**a**) TNF-α and (**b**) IL-1β concentrations (pg/mg protein). Data are shown as mean ± SEM. **** *p* < 0.0001.

Hippocampal TNF-α concentrations differed significantly among the experimental groups (F(2,21) = 36.13, *p* < 0.0001, R^2^ = 0.7748) (Figure 4a). Variance homogeneity was acceptable according to Brown–Forsythe (*p* = 0.3175) and Bartlett’s (*p* = 0.0187) tests. Post hoc analysis showed that LPS administration markedly increased the TNF-α levels compared with the Control group (183.8 ± 13.8 pg/mg vs. 77.5 ± 13.8 pg/mg, *p* < 0.0001). Carvacrol treatment significantly reduced the TNF-α concentrations relative to the LPS group (94.3 ± 13.8 pg/mg vs. 183.8 ± 13.8 pg/mg, *p* < 0.0001), restoring values close to the control levels.

Similarly, hippocampal IL-1β concentrations showed a highly significant difference among groups (F(2,21) = 40.45, *p* < 0.0001, R^2^ = 0.7939) (Figure 4b). LPS exposure markedly elevated the IL-1β levels compared with the Control (166.0 ± 15.1 pg/mg vs. 42.4 ± 15.1 pg/mg, *p* < 0.0001), while carvacrol treatment significantly attenuated this increase (64.6 ± 15.1 pg/mg vs. 166.0 ± 15.1 pg/mg, *p* < 0.0001).

Together, these results indicate that LPS-induced neuroinflammation was characterized by elevated hippocampal TNF-α and IL-1β levels, both of which were effectively suppressed by carvacrol administration, suggesting a strong anti-inflammatory and neuroprotective effect. Collectively, the behavioral, prostaglandin, and cytokine findings support the hypothesis that carvacrol mitigates LPS-induced hippocampal inflammation and cognitive impairment by modulating the prostaglandin–cytokine signaling pathways. These results form the basis for the subsequent discussion of the potential mechanisms underlying carvacrol’s neuroprotective actions.

## 4. Discussion

Neuroinflammation plays a central role in the development and progression of various neurodegenerative and neuropsychiatric disorders, including Alzheimer’s disease, Parkinson’s disease, and multiple sclerosis [18]. The LPS-induced model of neuroinflammation is a well-established paradigm to study systemic immune activation and cytokine-mediated neurotoxicity that lead to hippocampal dysfunction [19,20]. In line with previous evidence, LPS administration in the present study produced a robust neuroinflammatory response characterized by increased hippocampal PGE_2_, PGF_2_α, TNF-α, and IL-1β levels, accompanied by cognitive impairment in the Y-maze task. These findings confirm that peripheral immune challenge with LPS induces central inflammatory cascades that disrupt hippocampal-dependent memory functions [19].

In the open-field test, LPS and carvacrol treatment did not significantly alter locomotor activity, freezing time, or mobility rate, indicating that neither systemic inflammation nor drug administration affected general motor performance. In one study, adult mice that received i.p. LPS during the neonatal period showed no significant change in spontaneous motility in the open field test [21]. This finding ensures that the behavioral changes observed in the Y-maze test reflect true cognitive alterations rather than locomotor deficits. In one study, it was stated that LPS application only impaired memory performance without affecting functional motor parameters, and that in such models, motor changes should be distinguished from behavioral changes [22].

In the Y-maze task, LPS administration significantly reduced novel arm entries, suggesting impaired spatial recognition or working memory performance. In a study using an LPS-induced neurodegeneration model, it was reported that Y-maze performance decreased with LPS [23]. Although the carvacrol-treated animals showed slightly higher entry counts than the LPS group, this improvement was not statistically significant, indicating only a partial behavioral recovery. Interestingly, the time spent in the novel arm also decreased after carvacrol treatment, suggesting that the compound did not enhance, and may even have attenuated, exploratory drive in this paradigm. However, the total arm entries were comparable among groups, further confirming the absence of locomotor impairment.

Despite these mixed exploratory outcomes, the discrimination index was profoundly reduced by LPS and markedly restored by carvacrol treatment. This result implies that while carvacrol may not fully normalize exploratory behavior, it significantly ameliorates LPS-induced cognitive dysfunction. The improvement in discrimination performance likely reflects the compound’s ability to modulate hippocampal inflammatory and oxidative processes rather than motor activity per se. In line with the results of our study, carvacrol was reported to improve the discrimination index in LPS-induced descriptive memory impairments [24,25].

LPS administration induced a marked elevation in hippocampal PGE_2_, indicating strong activation of the COX-dependent prostanoid pathway [19,26]. Carvacrol significantly attenuated this increase, suggesting that its neuroprotective effect may involve the inhibition of COX-2 or mPGES-1 activity and subsequent PGE_2_ synthesis [27]. Since PGE_2_ acts through EP receptors to promote NF-κB-dependent cytokine release, its suppression by carvacrol likely contributes to the observed reduction in TNF-α and IL-1β levels [27,28].

Unlike PGE_2_, hippocampal PGD_2_ concentrations remained unchanged following LPS or carvacrol treatment. This selective regulation suggests that LPS preferentially activates the pro-inflammatory PGE_2_ pathway rather than globally influencing prostaglandin metabolism [19]. The stability of the PGD_2_ levels may also indicate a balanced response between pro- and anti-inflammatory prostaglandin signaling within the hippocampus. In contrast, the PGF_2_α concentrations were significantly elevated by LPS, reflecting enhanced lipid peroxidation and oxidative stress. Although carvacrol tended to lower the PGF_2_α levels, this effect did not reach statistical significance, indicating a partial modulation of prostanoid synthesis.

The cytokine profile further confirmed a robust neuroinflammatory response, with the TNF-α and IL-1β levels markedly increased after LPS exposure [5,29]. Carvacrol treatment significantly suppressed both cytokines, aligning with its known inhibitory effect on microglial activation and NF-κB-mediated transcriptional activity [11]. By attenuating these pro-inflammatory mediators, carvacrol likely prevents secondary neuronal injury and synaptic dysfunction in the hippocampus.

Beyond its anti-inflammatory effects, carvacrol possesses strong antioxidant and antiapoptotic properties that may contribute to its neuroprotective actions [24,30]. Excessive ROS generation during neuroinflammation amplifies cytokine signaling and impairs neuronal function [31]. Carvacrol has been shown to activate the Nrf2/HO-1 pathway, enhancing endogenous antioxidant defenses such as SOD and CAT, while concurrently reducing lipid peroxidation [32,33]. Furthermore, its ability to inhibit caspase-3 activation and Bax expression confers protection against apoptosis, maintaining neuronal integrity and synaptic stability under inflammatory stress [34,35]. These complementary mechanisms may explain the observed behavioral improvement despite partial effects on exploratory parameters.

The present study provides novel evidence that carvacrol simultaneously modulates prostaglandin and cytokine cascades in the hippocampus—an integrated regulatory mechanism not commonly reported for other phytochemicals such as curcumin or resveratrol [36,37]. By attenuating both PGE_2_ synthesis and downstream cytokine production, carvacrol disrupts the positive feedback loop that amplifies neuroinflammation through EP receptor–NF-κB coupling. This dual modulation may yield broader neuroprotective efficacy compared with single-pathway inhibitors. The observed behavioral recovery in the discrimination index further supports the functional relevance of this molecular regulation. Collectively, these findings expand the pharmacological profile of carvacrol and highlight its potential as an adjunctive therapeutic candidate for inflammation-associated cognitive disorders.

It should also be noted that the present study employed an acute LPS-induced neuroinflammation model, which primarily reflects short-term inflammatory responses rather than the progressive and multifactorial pathology of chronic neurodegenerative disorders. Therefore, the findings should be interpreted cautiously with respect to translational relevance to diseases such as Alzheimer’s disease or Parkinson’s disease. Future studies using chronic neurodegeneration models are needed to better clarify the long-term neuroprotective and anti-inflammatory potential of carvacrol.

Despite these promising results, some limitations must be acknowledged. The relatively small sample size and use of an acute LPS model may limit extrapolation to chronic neurodegenerative conditions. Future studies using prolonged exposure models could better clarify the sustained effects of carvacrol on neuroinflammation and memory. In addition, histopathological and immunohistochemical investigations were not performed in the present study. Evaluation of neuronal integrity, microglial activation, and astrocytic responses using markers such as COX-2, mPGES-1, Iba-1, GFAP, or NeuN would provide deeper insight into the cellular mechanisms underlying the neuroprotective effects of carvacrol and strengthen the interpretation of the biochemical findings. While the current findings suggest involvement of the NF-κB and Nrf2/HO-1 pathways, these mechanisms warrant direct experimental confirmation. Further work exploring dose dependency, pharmacokinetics, and synergistic interactions with other natural anti-inflammatory agents could support the translational application of carvacrol in the management of neuroinflammation-related cognitive impairment.

## 5. Conclusions

In this study, we demonstrated that acute systemic LPS administration induces a robust neuroinflammatory response in the hippocampus, characterized by increased PGE_2_, PGF_2_α, TNF-α, and IL-1β levels and accompanied by marked impairment in hippocampal-dependent spatial recognition memory. Importantly, these cognitive deficits occurred in the absence of locomotor alterations, indicating that the behavioral impairments primarily reflect disrupted mnemonic processes rather than reduced motor performance.

Carvacrol treatment effectively mitigated key aspects of this LPS-induced pathology. Specifically, carvacrol normalized the hippocampal PGE_2_ concentrations and significantly reduced the TNF-α and IL-1β levels while partially modulating PGF_2_α and markedly improved the discrimination index in the Y-maze task. These findings suggest that carvacrol exerts neuroprotective effects by targeting the prostaglandin–cytokine axis and dampening EP receptor–NF-κB-driven inflammatory signaling. In addition, its reported activation of Nrf2/HO-1-mediated antioxidant defenses and inhibition of apoptotic pathways likely contributes to the preservation of neuronal integrity and synaptic function under inflammatory stress.

## Data Availability

All data from this study are in the manuscript; please contact the corresponding author if further information is required.

## Supplementary Materials

The following supporting information can be downloaded at: https://www.mdpi.com/article/10.3390/biomedicines14071428/s1, Supplementary Figure S1. Body weight and body temperature during the experimental period. (a) Daily body weight measurements over 7 days and (b) terminal rectal temperature at euthanasia. Data are presented as mean ± SEM. ns: non-significant.
