# Supplementary figures and images for: Carvacrol Modulates the Hippocampal Prostaglandin–Cytokine Axis in LPS-Induced Neuroinflammation
Source: Biomedicines. 2026 Jun 24;14(7):1428. doi: 10.3390/biomedicines14071428 (PMC13405818; doi:10.3390/biomedicines14071428)

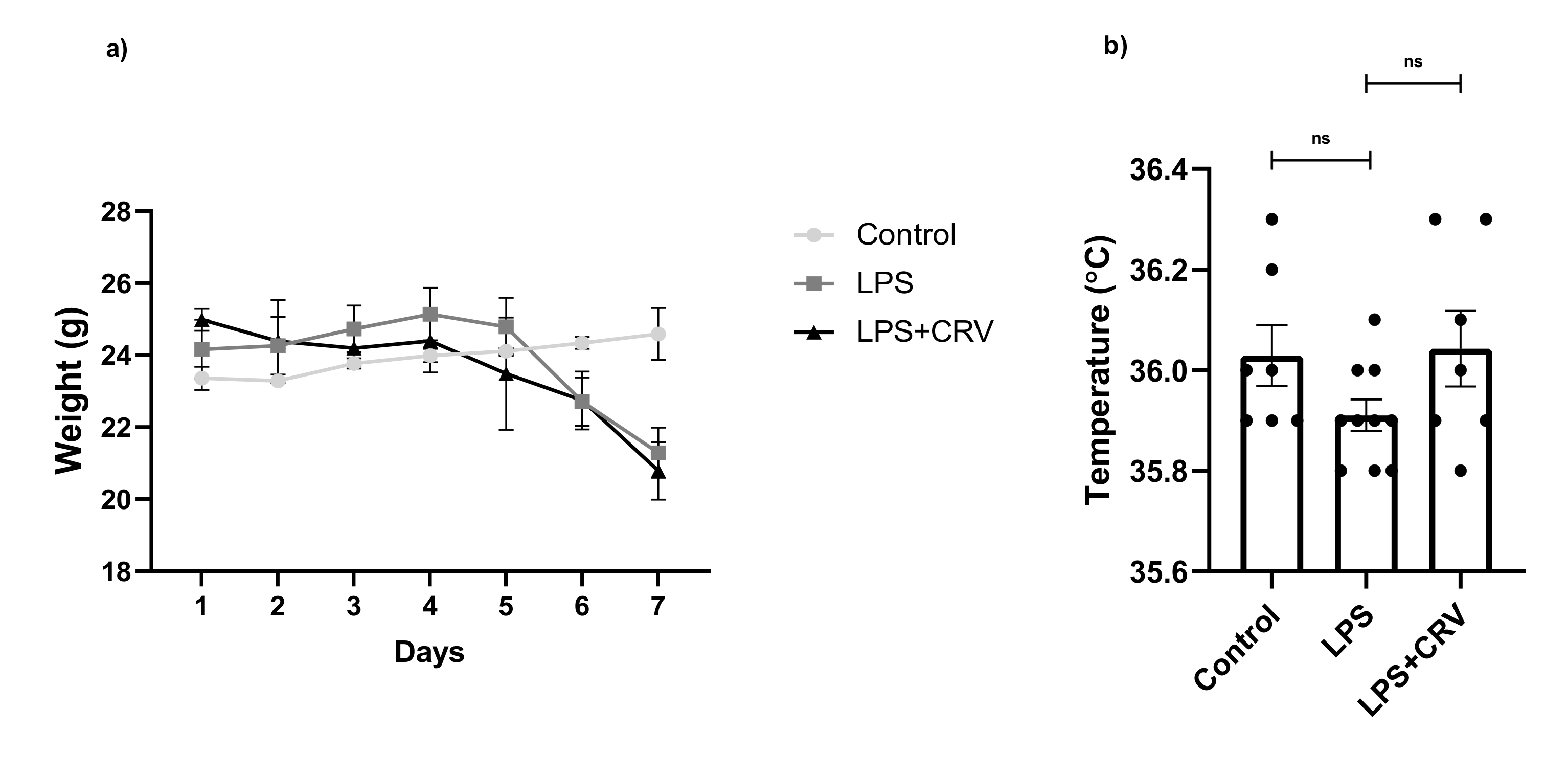

Supplement: Supplementary file 1 [file biomedicines-14-01428-s001.zip › biomedicines-4301505-supplementary.jpg]
